# Occurrence of *Mycoplasma* spp. in wild birds: phylogenetic analysis and potential factors affecting distribution

**DOI:** 10.1038/s41598-021-96577-0

**Published:** 2021-08-23

**Authors:** Anna Sawicka-Durkalec, Olimpia Kursa, Łukasz Bednarz, Grzegorz Tomczyk

**Affiliations:** 1 Department of Poultry Diseases, National Veterinary Research Institute, Aleja Partyzantów 57, 24-100 Puławy, Poland; 2 Bird Horizons Foundation, Spółdzielcza 34, 24-220 Niedrzwica Duża, Poland

**Keywords:** Infectious-disease diagnostics, Ecological epidemiology

## Abstract

Different *Mycoplasma* species have been reported in avian hosts. However, the majority of studies focus on one particular species of *Mycoplasma* or one host. In our research, we screened a total of 1141 wild birds representing 55 species, 26 families, and 15 orders for the presence of mycoplasmas by conventional PCR based on the 16S rRNA gene. Selected PCR products were sequenced to perform the phylogenetic analysis. All mycoplasma-positive samples were tested for *M. gallisepticum* and *M. synoviae*, which are considered the major pathogens of commercial poultry. We also verified the influence of ecological characteristics of the tested bird species including feeding habits, habitat types, and movement patterns. The presence of *Mycoplasma* spp. was confirmed in 498 birds of 29 species, but none of the tested birds were positive for *M. gallisepticum* or *M. synoviae*. We found possible associations between the presence of *Mycoplasma* spp. and all investigated ecological factors. The phylogenetic analysis showed a high variability of *Mycoplasma* spp.; however, some clustering of sequences was observed regarding particular bird species. We found that wild migratory waterfowl, particularly the white-fronted goose (*Anser albifrons*) and mallard (*Anas platyrhynchos*) could be reservoirs and vectors of mycoplasmas pathogenic to commercial waterfowl.

## Introduction

Mycoplasmas are the smallest bacteria widespread in nature. The *Mycoplasma* genus contains more than 100 species. Some of them appear to be more pathogenic than others but most of them occur as commensals or opportunistic pathogens. Various clinical signs and lesions may be caused by mycoplasmal infections, and these infections may lead to respiratory and reproductive disorders, conjunctivitis, arthritis, and skeletal abnormalities^1^.

The two well-known poultry mycoplasmas that are considered the most pathogenic, *Mycoplasma gallisepticum* (MG) and *Mycoplasma synoviae* (MS), have been detected many times in different wild bird species representing different orders. It is well known that many mycoplasmas are host-specific, therefore infections caused by MG and MS mainly manifest clinical signs in wild species of Galliformes^2–9^. The outbreaks of MG infection in wild passerines in the USA provide an example of its ability to adapt to a new host and to develop typical clinical signs of infection. Although MG was found in certain raptors^10^ and a broad range of species of the Passeriformes order^11^, the disease symptoms were found only in the *Fringillidae*^12–14^ and *Paridae*^15^ families. Conjunctivitis possibly due to MG infection has also been described in other avian species, but these data were based on observation of symptoms in birds and the presence of MG was not confirmed by diagnostic tests. Usually, asymptomatic hosts were considered vectors or reservoirs of MG^16^. A recent study proves that asymptomatic MG infection in the Eastern bluebird (*Sialia sialis*) can decrease the level of hemoglobin and body weight^17,18^. Another *Mycoplasma* species that can cause conjunctivitis is *Mycoplasma sturni*, but its host range is limited to only wild birds. *Mycoplasma sturni* was identified for the first time in a European starling (*Sturnus vulgaris*) with severe bilateral conjunctivitis^19^ and also isolated from other passerine species such as the northern mockingbird (*Mimus polyglottos*), blue jay (*Cyanocitta cristata*)^20^, house finch (*Haemorhous mexicanus*)^21^, cliff swallow (*Petrochelidon pyrrhonota*)^22^, American crow (*Corvus brachyrhynchos*), and American robin (*Turdus migratorius*)^23^. All these species of birds showed clinical signs comparable to those present in the European starling. However, the presence of *M. sturni* was also confirmed in birds that did not show any clinical signs ^23^ and in those birds whose symptoms were due to MG infection^21^. Other mycoplasmas which can present respiratory signs in wild birds are *M. buteonis*, *M. corogypsi*, *M. falconis*, and *M. gypis*, that were found in raptors from the Falconiformes and Accipitriformes orders^24^. Black vultures (*Coragyps atratus*) infected with *Mycoplasma corogypsi* developed polyarthritis^25,26^. Erdélyi et al*.*^27^ reported a case of skeletal deformities in a Saker falcon nestling (*Falco cherrug*) that was associated with infection of *Mycoplasma buteonis*. This report highlights the possibility of the vertical spread of mycoplasmas in free-living falcon populations. Another study confirms that some *Mycoplasma* species isolated from raptors can be vertically transmitted via semen. Fischer et al*.*^28^ described a new species of mycoplasma, *Mycoplasma seminis*, that was isolated from semen of a gyrfalcon (*Falco rusticolus*) without any clinical signs of disease. Although mycoplasmas were isolated from raptors with clinical signs, many authors describe them as opportunistic pathogens or as a part of the physiological microbiota of the upper respiratory tract^29–31^.

More recent evidence highlights that the significance and knowledge of some *Mycoplasma* species may change. One of the best examples is *M. anserisalpingitis* (formerly known as *Mycoplasma* 1220), which was isolated from geese. Awareness of *M. anserisalpingitis* as a pathogen of commercial waterfowl has grown due to the development of new genetic techniques in recent years^32^. The number of publications reporting the occurrence of *M. anserisalpingitis* in different countries can be observed to increase^33–35^. However, the range of mycoplasma species important in the pathology of geese is still expanding, which is demonstrable by the reports of unknown novel *Mycoplasma* spp. that may cause phallic deformities in commercial flocks of goose breeders^36^. In the past, only the respiratory and synovitis forms of the disease were reported for MS infections, and the majority of them were subclinical. Nowadays, some of the MS strains reported worldwide also show tropism to the oviduct and may cause eggshell apex abnormalities and egg drops^37–39^. Knowledge of the pathogenicity of mycoplasmas to wild birds is limited and a complication to its elucidation is the potential for different strains of one species of *Mycoplasma* to have different levels of pathogenicity^40,41^. Additionally, clinical signs of infection by *Mycoplasma* may only appear under specific stress conditions or when birds’ immunity is impaired^42^.

For decades, scientists have been trying to establish the role of wild birds as possible vectors for transmission of mycoplasmas to commercial poultry. Published papers mainly concern the occurrence of MG and MS where synanthropic birds are recognized as mechanical carriers of these pathogens^43–47^. However, in the context of transmission of *Mycoplasma* species pathogenic to commercial waterfowl, the literature data is not sufficient.

The objectives of our study were: (1) to survey a large number of wild birds from different orders, families and species for the occurrence of *Mycoplasma* spp.; (2) to verify the influence of feeding habits, habitat type and movement patterns of particular bird species on the occurrence of *Mycoplasma* spp.; (3) to verify the presence of MG and MS in wild birds; and (4) to analyze the phylogeny of selected *Mycoplasma* spp.-positive samples.

## Results

### Occurrence of *Mycoplasma* spp. in wild birds

The results of the occurrence of *Mycoplasma* spp. in different wild bird species are presented in Table 1. Raw data are available in the Supplementary Dataset. *Mycoplasma* spp. were found in the samples of 498 birds representing 29 species. Bacterial DNA was found in 462 oropharyngeal swab samples of 26 species, in 43 cloacal swab samples of 11 species, and in both oropharyngeal and cloacal swabs in 6 birds of 3 species. A high occurrence of *Mycoplasma* spp. was found in a variety of species; namely, the common gull (*Larus canus*) (N = 111; 97.3%), European herring gull (*Larus argentatus*) (N = 16; 93.8%), common kestrel (*Falco tinnunculus*) (N = 15; 93.3%), feral pigeon (*Columba livia domestica*) (N = 13; 92.3%), black-headed gull (*Chroicocephalus ridibundus*) (N = 84; 79.8%), white stork (*Ciconia ciconia*) (N = 66; 78.8%;), lesser spotted eagle (*Clanga pomarina*) (N = 28; 64.3%), great cormorant (*Phalacrocorax carbo*) (N = 37; 51.4%), and mallard (*Anas platyrhynchos*) (N = 326; 36.8%). We found differences in the occurrence of Mycoplasma spp. between oropharyngeal and cloacal swabs in mute swan (*Cygnus olor*) (p = 0.011) and white storck (p = 8.7 × 10^–8^). All of the *Mycoplasma* spp.-positive samples were found to be negative for MG or MS.

**Table 1:** Birds tested for the presence of *Mycoplasma* spp.

Order / family and species	Diet; habitat; movement pattern^a^	Oropharyngeal swabs		Cloacal swabs		All birds tested	
		Positive /N	% (95% CI)	Positive /N	% (95% CI)	Positive /N	% (95% CI)
**Accipitriformes / Accipitridae**							
Common buzzard (*Buteo buteo*)	AB; TR; M	1 / 4	–	2 / 4	–	3 / 5	–
Golden eagle (*Aquila chrysaetos*)	AB; TR; M	0 / 2	–	0 / 4	–	0 / 4	–
Lesser spotted eagle (*Clanga pomarina*)	AB; TR; M	18 / 28	64.3 (45.8 –79.3)	–	–	18 / 28	64.3 (45.8 –79.3)
Short-toed snake eagle (*Circaetus gallicus*)	AB; TR; M	0 / 1	–	–	–	0 / 1	–
Western marsh harrier (*Circus aeruginosus*)	AB; AQ; M	1 / 1	–	–	–	1 / 1	–
White-tailed eagle (*Haliaeetus albicilla*)	AB; AQ; S	3 / 3	–	–	–	3 / 3	–
**Anseriformes / Anatidae**							
Eurasian teal (*Anas crecca*)	MX; AQ; M	1 / 5	–	3 / 21	14.3 (5 –34.6)	4 / 26	15.4 (6.2 –33.5)
Garganey (*Spatula querquedula*)	MX; AQ; M	0 / 2	–	–	–	0 / 2	–
Graylag goose (*Anser anser*)	PB; AQ; M	2 / 4	–	2 / 4	–	2 / 4	–
Greater white-fronted goose (*Anser albifrons*)	PB; AQ; M	0 / 2	–	2 / 2	–	2 / 2	–
Long-tailed duck (*Clangula hyemalis*)	MX; AQ; M	0 / 1	–	–	–	0 / 1	–
Mallard (*Anas platyrhynchos*)	MX; AQ; M	102 / 291	35.1 (29.8 –40.7)	19 / 40	47.5 (32.9 –62.5)	120 / 326	36.8 (31.8–42.2)
Mute swan (*Cygnus olor*)	PB; AQ; S	17 / 151	11.3 (7.1 –17.3)	8 / 25	32 (17.2 –51.6)	25 / 167	15 (10.4–21.2)
Taiga bean goose (*Anser fabalis*)	PB; AQ; M	0 / 1	–	1 / 1	–	1 / 1	–
Velvet scoter (*Melanitta fusca*)	MX; AQ; M	0 / 4	–	–	–	0 / 4	–
Whooper swan (*Cygnus cygnus*)	PB; AQ; M	0 / 1	–	–	–	0 / 1	–
**Apodiformes / Apodidae**							
Common swift (*Apus apus*)	AB; TR; M	3 / 3	–	–	–	3 / 3	–
**Charadriiformes / Alcidae**							
Common murre (*Uria aalge*)	AB; AQ; M	0 / 1	–	–	–	0 / 1	–
Razorbill (*Alca torda*)	AB; AQ; M	1 / 1	–	–	–	1 / 1	–
**Charadriiformes / Laridae**							
Black-headed gull (*Chroicocephalus ridibundus*)	MX; AQ; M	67 / 75	89.3 (80.3 –94.5)	0 / 9	–	67 / 84	79.8 (70–87)
Common gull (*Larus canus*)	MX; AQ; M	108 / 111	97.3 (92.4 –99.1)	–	–	108 / 111	97.3 (92.4–99.1)
Common tern (*Sterna hirundo*)	AB; AQ; M	0 / 2	–	0 / 2	–	0 / 3	-
European herring gull (*Larus argentatus*)	AB; AQ; M	15 / 16	93.8 (71.7 –98.9)	–	–	15 / 16	93.8 (71.7–98.9)
**Ciconiiformes / Ciconiidae**							
White stork (*Ciconia ciconia*)	AB; TR; M	51 / 64	79.7 (68.3 –87.7)	4 / 24	16.7 (6.7–35.9)	52 / 66	78.8 (67.5–86.9)
**Columbiformes / Columbidae**							
Common wood pigeon (*Columba palumbus*)	PB; TR; M	0 / 2	–	–	–	0 / 2	–
Feral pigeon (*Columba livia domestica*)	PB; TR; S	12 / 13	92.3 (66.7 –98.6)	–	–	12 / 13	92.3 (66.7 –98.6)
**Falconiformes / Falconidae**							
Common kestrel (*Falco tinnunculus*)	AB; TR; S	14 / 15	93.3 (70.2 – 98.8)	–	–	14 / 15	93.3 (70.2 –98.8)
**Gaviiformes / Gaviidae**							
Red -throated loon (*Gavia stellata*)	AB; AQ; M	1 / 1	–	–	–	1 / 1	–
**Gruiformes / Gruidae**							
Common crane (*Grus grus*)	MX; AQ; M	0 / 1	–	0 / 1	–	0 / 1	–
**Gruiformes / Rallidae**							
Eurasian coot (*Fulica atra*)	MX; AQ; M	4 / 4	–	–	–	4 / 4	–
**Passeriformes / Corvidae**							
Common raven (*Corvus corax*)	AB; TR; S	0 / 1	–	0 / 2	–	0 / 2	–
Eurasian jay (*Garrulus glandarius*)	MX; TR; S	0 / 3	–	0 / 1	–	0 / 3	-
Eurasian magpie (*Pica pica*)	MX; TR; S	0 / 2	–	–	–	0 / 2	-
Hooded crow (*Corvus cornix*)	MX; TR; S	0 / 1	–	–	–	0 / 1	-
Rook (*Corvus frugilegus*)	MX; TR; S	0 / 1	–	–	–	0 / 1	–
Western jackdaw (*Corvus monedula*)	MX; TR; S	0 / 6	–	–	–	0 / 6	–
**Passeriformes / Emberizidae**							
Yellowhammer (*Emberiza citrinella*)	PB; TR; S	4 / 8	–	–	–	4 / 8	–
**Passeriformes / Fringillidae**							
Common chaffinch (*Fringilla coelebs*)	PB; TR; M	2 / 5	–	–	–	2 / 5	–
Common redpoll (*Acanthis flammea*)	PB; TR; M	3 / 51	5.9 (2 – 15.9)	–	–	3 / 51	5.9 (2 –15.9)
Eurasian siskin (*Spinus spinus*)	PB; TR; M	0 / 51	0 (0 – 7)	–	–	0 / 51	0 (0 –7)
European greenfinch (*Chloris chloris*)	PB; TR; S	0 / 9	–	–	–	0 / 9	–
**Passeriformes / Muscicapidae**							
European robin (*Erithacus rubecula*)	AB; TR; M	1 / 2	–	–	–	1 / 2	–
Passeriformes / Paridae							
Eurasian blue tit (*Cyanistes caeruleus*)	AB; TR; S	1 / 3	–	–	–	1 / 3	–
Great tit (*Parus major*)	AB; TR; S	7 / 22	31.8 (16.4 – 52.7)	–	–	7 / 22	31.8 (16.4 –52.7)
**Passeriformes / Passeridae**							
Eurasian tree sparrow (*Passer montanus*)	PB; TR; S	0 / 2	–	–	–	0 / 2	–
**Passeriformes / Sittidae**							
Eurasian nuthatch (*Sitta europaea*)	AB; TR; S	0 / 1	–	–	–	0 / 1	–
**Passeriformes / Sturnidae**							
Common starling (*Sturnus vulgaris*)	MX; TR; S	0 / 2	–	–	–	0 / 2	–
**Passeriformes / Sylviidae**							
Eurasian blackcap (*Sylvia atricapilla*)	AB; TR; S	0 / 1	–	–	–	0 / 1	–
**Passeriformes / Turdidae**							
Common blackbird (*Turdus merula*)	MX; TR; M	4 / 12	33.3 (13.8 – 60.9)	–	–	4 / 12	33.3 (13.8 –60.9)
Song thrush (*Turdus philomelos*)	MX; TR; M	0 / 11	0 (0 – 25.9)	1 / 6	–	1 / 11	9.1 (1.6 –37.7)
**Pelecaniformes / Ardeidae**							
Grey heron (*Ardea cinerea*)	AB; AQ; S	0 / 4	–	–	–	0 / 4	–
**Piciformes / Picidae**							
Great spotted woodpecker (*Dendrocopos major*)	MX; TR; S	0 / 5	–	–	–	0 / 5	–
**Podicipediformes / Podicipedidae**							
Great crested grebe (*Podiceps cristatus*)	AB; AQ; M	0 / 1	–	–	–	0 / 1	–
**Strigiformes / Strigidae**							
Tawny owl (*Strix aluco*)	AB; TR; S	0 / 2	–	0 / 2	–	0 / 2	–
**Suliformes / Phalacrocoracidae**							
Great cormorant (*Phalacrocorax carbo*)	AB; AQ; M	19 / 37	51.4 (35.9 – 66.6)	–	–	19 / 37	51.4 (35.9 –66.6)

^a^Guilds: *AB* animal -based diet, *PB* plant -based diet, *MX* mixed diet, *AQ* aquatic habitat, *TR* terrestrial habitat, *M* migratory, *S* sedentary.

### Potential factors affecting occurrence of *Mycoplasma* spp.

**Figure 1:**
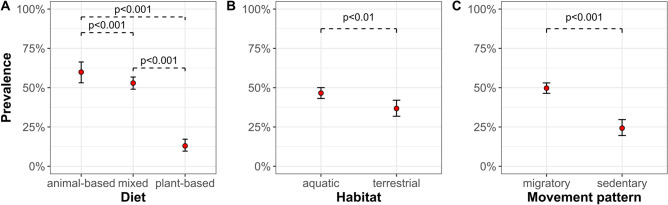
The prevalence of *Mycoplasma* spp. in different species of wild birds by their dietary preferences (**A**), habitat types (**B**), and movement patterns (**C**). Differences are significant at p < 0.05 (Fisher’s exact test).

We found that the prevalence of *Mycoplasma* spp. was related to the characteristics of particular species of birds and chiefly their feeding habits, movement patterns, and habitat types. Our results showed the highest prevalence of *Mycoplasma* spp. in species that eat an animal-based diet (N = 207; 59.9%; 95% CI 53.1–66.3), lower prevalence in those sustained by a mixed diet (N = 633; 52.9; 95% CI 49.0–56.8), and the lowest in birds feeding on plant material (N = 301; 13%; 95% CI 9.6–17.2) (Fig. 1A). Our results revealed that the prevalence of these bacteria in birds inhabiting aquatic environments (N = 801; 46.6%; 95% CI 43.1–50.0) was higher than in the terrestrial ones (N = 340; 36.8%; 95% CI 31.8–42.0) (Fig. 1B). Significant differences were also found in the prevalence of *Mycoplasma* spp. between migratory (N = 869; 46.8%; 95% CI 46.4–53.0) and sedentary birds (N = 272; 24.3%; 95% CI 19.6–29.7) (Fig. 1C).

### Molecular characteristics of detected *Mycoplasma* spp.

**Figure 2:**
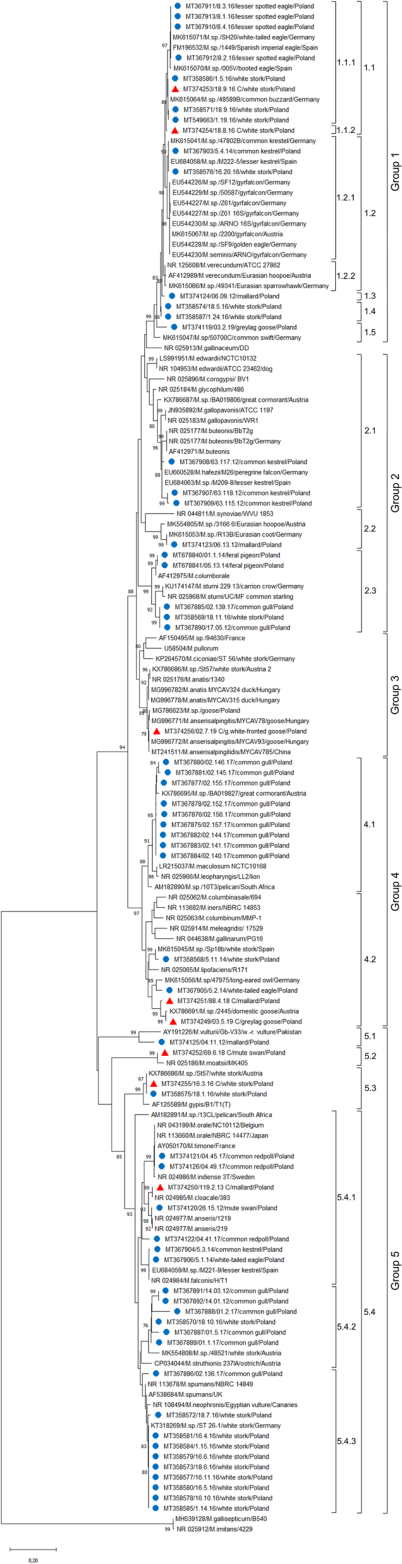
Maximum likelihood phylogenetic tree showing the relationships between 66 *Mycoplasma* sp. sequences detected in this study and 78 *Mycoplasma* sp. sequences retrieved from GenBank based on 16S rRNA gene sequences. The sequences of *M. gallisepticum* and *M. imitans* were used as the out-group. Bootstrap was conducted with 1000 repetitions. Red triangles represent cloacal swabs and blue circles oropharyngeal swabs.

We performed a phylogenetic analysis of 66 selected sequences. The phylogenetic tree is shown in Fig. 2. The recently proposed signature index for the identification of phylogenetic placement revealed that the sequences obtained were within the *M. synoviae* and *M. hominis* clusters, both forming part of the hominis phylogenetic group of the *Mycoplasma* genus. Wild bird mycoplasmas formed five main groups. No strong consistent pattern was observed for associations between wild bird hosts and *Mycoplasma* species; however, some clustering of sequences was observed. Group 1 included sequences from birds of prey, white storks, and waterfowl and was divided into five subgroups, the first of which (1.1) could be divided into two sub-branches. The first sub-branch, 1.1.1, contained isolates from cloacal and oropharyngeal samples from white storks (GenBank accession nos MT358571, MT358586, MT367910, MT367911, MT367912, MT367913, MT374253, and MT549663), as well as sequences isolated from oropharyngeal samples from several raptor species which were closely related (98.5–100% similarity) to mycoplasmas detected in raptors from Germany and Spain (GenBank accession nos FM196532, MK615064, MK615070, and MK615071). However, we also found one sequence (sub-branch 1.1.2) originating from a white stork (GenBank accession no. MT374254) which had lower similarity to sequences from the first sub-branch. The amplicons in this second sub-branch were found in the trachea of a white stork and common kestrel (GenBank accession nos MT358576 and MT367903) and were similar to different *Mycoplasma* spp. (GenBank accession nos MK615067, EU544226, EU544227, EU544228, EU544229), and *M. seminis* (GenBank accession no. EU544230) detected in birds of prey from other European countries. We found four genotypes (subgroups 1.3–1.5) that showed low similarity to other sequences from the GenBank database (≤ 97%).

The second main branch, Group 2, includes three subgroups. The analysis of subgroup 2.1 revealed that three strains isolated from common kestrels (GenBank accession nos MT367907, MT367908, and MT367909) were closely related (98.4–99.3% similarity) to strains of *Mycoplasma hafezii* isolated from a peregrine falcon (*Falco peregrinus*) from Germany (GenBank accession no. EU660528) and a lesser kestrel (*Falco naumanni*) from Spain (GenBank accession no. EU684063). The sequence incorporated in the subgroup 2.2 was obtained from a mallard (GenBank accession no. MT374123) and showed similarity to a German isolate from a Eurasian coot (*Fulica atra*) (GenBank accession no. MK615053). The third subgroup (2.3) comprised Polish strains isolated from feral pigeons (GenBank accession nos MT678840 and MT678841), common gulls (GenBank accession nos MT367885 and MT367890), and a white stork (GenBank accession no. MT358569). The nucleotide sequence homology within this cluster ranged from 95.3 to 100%. The Polish sequences isolated from the pigeons had 100% similarity to *M. columborale* (GenBank accession no. AF412975). Strong sequence identity was also observed between isolates of *M. sturni* (GenBank accessions nos KU174147 and NR025968) and isolates from common gulls and white storks.

To the third group only one sequence of *Mycoplasma* spp. was classified. This sequence was obtained from a white-fronted goose (*Anser albifrons*) and showed identity with previously published *M anserisalpingitis* sequences from Hungarian (Genbank accession no. MG996772), Chinese (MT241511), and Polish domestic geese (MG786623).

Group 4 showed a high level of genetic variability, with two clearly distinguishable subgroups. Subgroup 4.1 included sequences obtained from common gulls (GenBank accession nos MT367875, MT367876, MT367877, MT367878, MT367880, MT367881, MT367882, MT367883, and MT367884) that were similar to Austrian sequences from a great cormorant (GenBank accession no. KX786695), whereas 4.2 contained sequences from cloacal swabs collected from a graylag goose (*Anser anser*) and mallard (GenBank accession nos MT374249 and MT374251) that were similar to sequences of *Mycoplasma* spp. isolated from a domestic goose from Austria. Additionally, subgroup 4.2 included sequences obtained from a Polish white-tailed eagle (*Haliaeetus albicilla*) (GenBank accession no. MT367905) which was similar to mycoplasma sequenced from a long-eared owl (*Asio otus*) (GenBank accession no. MK615056) and white storks from Poland and Spain (GenBank accession nos MT358568 and MK615045).

*Mycoplasma* spp. samples of the second clade formed a distinct group including four subgroups (5.1–5.4). The first two subgroups contained an oropharyngeal sample from a mallard (GenBank accession no. MT374125) identical to *M. vulturii* (GenBank accession no. AY191226) detected in an Oriental white-backed vulture (*Gyps bengalensis*) from Pakistan and also contained a cloacal sample from a mute swan (*Cygnus olor*) (GenBank accession no. MT374252) that was identical to *M. moatsii* (GenBank accession no. NR025186). The third subgroup (5.3) comprised sequences from two Polish (GenBank accession nos MT358575 and MT374255) and one Austrian (GenBank accession no. KX786686) white stork and were slightly related to *M. gypis* (97% similarity). The fourth subgroup (5.4) could be divided into three sub-branches and showed a high level of genetic variability. However, strong consistent associations between infected host species and detected *Mycoplasma* species could be observed within this subgroup. The subbranch 5.4.1 included isolates from Polish birds of prey (GenBank accession nos MT367904 and MT367906) which revealed 100% sequence similarity to *M. falconis* (GenBank accession no. NR024984) and one isolate of *Mycoplasma* sp. from a lesser kestrel from Spain (GenBank accession no. EU684059). The sequences of *M. anseris* and *M. cloacale* were clustered with sequences obtained from Polish wild waterfowl (GenBank accession nos MT374120 and MT374250). The remaining two sequences from common redpolls (*Acanthis flammea*) (GenBank accession nos MT374121 and MT374126) showed identity with *M. orale* (GenBank accession no. NR113660). The homology within nucleotide sequences of the next sub-branch (5.4.2) ranged from 95 to 100% and this grouping contained oropharyngeal samples from white storks and common gulls (GenBank accession nos MT358570, MT367886, MT367887, MT367888, MT367889, MT367891, and MT3678892). The last sub-branch (5.4.3) contained oropharyngeal samples from white storks (GenBank accession nos MT358572, MT358573, MT358577, MT358578, MT358579, MT358580, MT358581, MT358584, and MT358585) related to a German isolate of *Mycoplasma* sp. (GenBank accession no. KT318269) as well as *M. spumans* (GenBank accession nos NR113678 and AF538684), *M. neophronis* (GenBank accession no. NR108494) and *M. struthionis* (GenBank accession no. CP034044). The similarities among sequences of this subgroup were within the range of 97–100%.

## Discussion

### Occurrence of *Mycoplasma* spp. in wild birds

In general, mycoplasmas tend to exhibit host specificity and tissue tropism. Some species show more diverse tissue tropism due to differences between strains (e.g. MS), but the majority of species exhibit a predilection for selected anatomical sites^48^. For mycoplasmas typical for poultry, the sites of swab sampling are well known. In our study, we tested swabs from the oropharynx and/or cloaca of different species of wild birds. Sampling from both sites gave us an opportunity to determine the site with the higher frequency of *Mycoplasma* spp.

The occurrence of *Mycoplasma* spp. other than MG in wild birds is poorly documented. Our findings showed a higher occurrence of other mycoplasma species in some orders of birds such as Charadriiformes, Ciconiiformes, Accipitriformes and Falconiformes than in Passeriformes and others. The high occurrence of *Mycoplasma* spp. in raptors observed in our study is congruent with results that were reported in lesser kestrels^49^ and a western marsh harrier from Germany^31^ as well as different raptor species in Illinois^10^. A previous study^50^ showed the presence of *Mycoplasma* spp. in the common buzzard (*Buteo buteo*) which is in complete agreement with our findings. As far as we know, our study is the first to describe the occurrence of *Mycoplasma* in two species of raptors: the lesser spotted eagle and white-tailed eagle.

Charadriiformes, especially the Laridae family, are known as reservoirs of numerous viral and bacterial pathogens. However, only a few reports describe the presence of mycoplasmas in gulls^51^. In our study, the prevalence of *Mycoplasma* spp. in all species of gulls was high (Table 1). This is in good agreement with the results of recent studies obtained with the use of next-generation sequencing (NGS), in which *Mycoplasma* genus bacteria were detected as the most predominant^52,53^. Our results obtained from clinically healthy birds support the view that *Mycoplasma* species could be a part of the normal microflora of gulls. The majority of feral pigeons were found to be *Mycoplasma* spp.-positive and this result is in line with previous studies from Japan^54^. Similar results were also obtained in racing and ornamental pigeons^55,56^. The high occurrence of *Mycoplasma* in white storks observed in our study corroborates the results reported by Möller Palau-Ribes et al*.*^57^ for a white stork population in Germany. We detected mycoplasmas in swabs from both sites in white storks, but their occurrence was lower in cloacal swab samples than in oropharyngeal ones. Samples collected from the Anseriformes order were the most abundant in our study, and 122 out of 462 (26.4%) oropharyngeal and 35 of 93 (37.6%) cloacal swab samples were found to be *Mycoplasma* spp. positive. Previous studies conducted in the USA by Goldberg et al*.*^58^ showed lower prevalence of *Mycoplasma* spp. in wild waterfowl (3.5% and 5.5% of tracheal swabs collected from live and dead birds, respectively). The discrepancy between our results and those reported by Goldberg et al*.* could be related to the method used for *Mycoplasma* spp. detection and differences in the geographical locations of tested birds. The presence of different mycoplasmas in wild waterfowl has been reported in Europe. Bradbury et al*.*^59^ found *M. cloacale* in tufted ducks (*Aythya fuligula*) and common pochards (*Aythya ferina*) from the United Kingdom. Another study performed in Spain by Poveda et al*.*^60^ describes the presence of *Mycoplasma anatis* in 3 out of 10 tested shovelers (*Anas clypeata*). However, the authors did not find any other mycoplasmas in tested mallards or Eurasian teals (*Anas crecca*), which was contradictory to our findings. However, the lack of mycoplasma-positive results in the Spanish study might be caused by the small number of birds tested and the different detection method. It is worth noting that we also found *Mycoplasma* spp. in different species of wild geese and in mute swans.

Our study, which covers a broad range of wild bird species, may help to deepen the understanding of the role of the tested species as *Mycoplasma* spp. reservoirs or vectors. Several studies have focused on wild birds as potential vectors of those species of *Mycoplasma* that are pathogenic to commercial poultry. To the best of our knowledge, our study is the first to reveal the role of wild geese as potential vectors of *Mycoplasma* strains that may pose a health risk to commercial waterfowl. We wanted to identify *Mycoplasma* spp. in different species of wild birds. Therefore, we used 16S rRNA gene sequences, which are considered to be an excellent choice for preliminary taxonomic classification and phylogenetic assignment of *Mycoplasma* isolates to certain groups^61^. It is worth emphasizing that our sequences originated directly from swab samples, which gave the possibility to detect numerous novel sequences that are phylogenetically assigned to the Mollicutes class and potentially represent novel uncharacterized *Mycoplasma* species. The 16S rRNA gene sequences have become one of the mandatory requirements for the description of new Mollicutes species. However, many additional analyses are required for the discovery and characterization of a novel species. The 16S rRNA genes have lower variability compared to the other genetic markers such as beta subunit (rpoB) genes or the 16S–23S rRNA intergenic transcribed spacer region (ITS)^62^.

The phylogenetic analysis showed identity between *Mycoplasma* spp. isolates obtained from two different groups of birds: white storks (MT358571, MT358576, MT358586, MT374253, and MT549663) and birds of prey (EU684058, MK615041, MK615064, and MT367903). This could be explained by those birds possibly sharing species of commensal mycoplasma^13,63^. The other sequences that were found by us in white storks showed similarity with German and Austrian isolates. However, none of the sequences collected in our study were similar to *M. ciconiae* sp. nov. which is considered to be common in the white stork population^57^. Möller Palau-Ribes et al*.* made a serological analysis to characterize the *M. ciconiae* and obtained a faint reaction with *M. sturni* antiserum. Surprisingly, the sequence found by us in Polish white storks (GenBank accession no. MT358569) was similar to that in *M. sturni* (96.6%), which could explain the positive reaction described in the aforementioned study. Our phylogenetic analyses also showed high similarity between sequences obtained from white storks and gulls in some clades. This finding revealed a potential role of the host diet in the frequency of detection of commensal *Mycoplasma* species. Furthermore, the dendrogram demonstrated that the sequences originating from gulls were clustered and those clusters were dispersed among clades. This may suggest that Laridae may be hosts of different mycoplasmas that could be unique to gulls. We believe that future work needs to be done to investigate and characterize the mycoplasmas that could be found in this family of birds.

The phylogenetic analysis of *Mycoplasma* spp. sequences obtained from birds of prey leads to the conclusion that most of them demonstrate close relatedness and specificity only for these hosts. Similar relationships were observed in wild waterfowl. The sequences of *Mycoplasma* spp. originating from mallards (MT374250 and MT374251), a mute swan (MT374120), and a graylag goose (MT374249) showed close similarity to mycoplasmas typically associated with Anseriformes. Our attention was drawn to the similarity of the sequence from a white-fronted goose (MT374256) to sequences of *M. anserisalpingitis* that were isolated from domestic geese from Hungary, China and Poland^34^. The results demonstrate that wild anserids can be reservoirs and vectors of various *Mycoplasma* species pathogenic to commercial waterfowl.

We have also identified a 100% sequence similarity between sequences obtained from common redpolls and *M. orale* (GenBank accession nos NR043199 and NR113660). This *Mycoplasma* species is considered a non-pathogenic or opportunistic part of the flora of the human upper respiratory tract^64^ and is well known as a common cell culture contaminant^65^. Microbiome analysis has revolutionized knowledge of the health consequences of transmission of non-pathogenic microbes between humans and animals. A recent review of the literature on this topic found significant consequences of the transfer of non-pathogenic microbes^66^. Furthermore, metagenomic studies have proved that bacteria of the human oral microbiota could possess antibiotic resistance genes^67–69^. It is crucial to note that antibiotic resistance genes can be transferred to bacteria of the same or different species. The presence of antibiotic resistance genes in bacteria isolated from wild animals may be associated with their proximity to human populations^70^. Wild birds which spend time close to human settlements may acquire bacteria with these genes and contribute to their widespread dissemination, especially if the birds are migratory^71,72^. Further work needs to be done to verify whether the common redpoll, as a synanthropic and migratory bird, could play a role as carrier or vector of antimicrobial-resistant strains of *Mycoplasma*.

Knowledge of the impact of feeding behavior on bacterial microbiota in wild birds is limited. There are only few elucidated examples of the relationship between feeding habits of birds and the occurrence of particular bacteria. A good example is the difference in occurrence of *Streptococcus* spp. between granivorous and omnivorous birds^73^. Another study documented the impact of diet diversity on richness of the microbiota in 13 different bird species from Slovenia^74^. Stenkat^75^ found that the presence of *Enterobacteriaceae* was significantly associated with an animal-based diet, especially a piscivorous one, while the presence of *Pseudomonadaceae* correlated positively with a herbivorous diet. Our findings revealed a higher occurrence of *Mycoplasma* spp. in birds that have animal-based diets than in herbivorous and omnivorous birds.

Habitat was verified as another potential factor which may have an influence on the occurrence of *Mycoplasma* spp. in birds. Our results show that the occurrence of *Mycoplasma* spp. was more frequent in birds inhabiting aquatic environments than in those living in terrestrial ones. The aquatic environment provides habitats to a wide range of bird species, for many of which it is the principal one, while for others it is only a feeding place. Some of them are gregarious, feeding side by side in mixed-species flocks, and leave a large amount of feces which also could be a source of *Mycoplasma* spp.^76^.

We found a high frequency of *Mycoplasma* spp. in migrating birds. The similarity of the *Mycoplasma* spp. sequence originating from white fronted geese to isolates of *M. anserisalpingitis* that were reported from other parts of the world confirmed that wild birds could play a potential role in the transmission and spread of mycoplasmas. The white-fronted goose is a migratory bird of which the breeding grounds cover almost the whole circumpolar Arctic^77^. However, these birds choose various wintering destinations such as eastern Asia^78^ or western Europe^79^. They make many stopovers during migration, which increases the risk of contamination of domestic goose water and pastures by their feces. Excluding an example of transmission of *M. gallisepticum* between house finch populations in the USA, no evidence of pathogenic mycoplasmas in migratory birds has been documented. The second reason that migration could have an influence on the occurrence of *Mycoplasma* spp. is the similarity between sequences from Polish white storks and those originating from Spanish imperial eagles (*Aquila adalberti*). The presence of the same *Mycoplasma* spp. in those two species of birds could be caused by their sharing of a wintering area^80,81^ as well as a probable diet.

In our study, we used a conventional PCR based on the 16S rRNA gene which might be considered either its potential or its limitation. We are aware that culture isolation is essential for the identification of the organism. However, the culture of unknown *Mycoplasma* species can be extremely hard due to the fastidious conditions required for their growth. Moreover, our aim was to detect different *Mycoplasma* species simultaneously, therefore we chose the PCR based on the 16S rRNA gene. The majority of *Mycoplasma* sequences in the GenBank database are sequences of that gene, which was helpful in the phylogenetic analysis of our results. As was mentioned above, the use of 16S rRNA helped us to detect sequences that potentially represent novel uncharacterized species. Another possible limitation of our work is the small sample size for some bird species. However, we decided to report these results, because in some cases, they were obtained from rare bird species.

In conclusion, the observed distribution of *Mycoplasma* spp. revealed that particular bird species have a predisposition to be hosts of these microorganisms. The identification of mycoplasmas detected in gulls and white storks suggested that they could be hosts of more than one species of *Mycoplasma*. We observed possible relationships between the presence of *Mycoplasma* spp. and the ecology of each bird species, i.e., its feeding habits, preferred habitats, and migration patterns. Further studies are necessary to verify the role of these mycoplasmas as opportunistic pathogens. Our results support the idea that wild migratory waterfowl could be a reservoir and vector of mycoplasmas pathogenic to commercial geese. Nevertheless, our results based on a wide range of tested bird species did not confirm the presence of MG or MS, thus the risk of transmission of those pathogens from wild birds to commercial poultry is relatively low.

## Materials and methods

### Birds

Samples of swabs were taken from 1141 wild birds representing 55 species, 26 families, and 15 orders between 2011 and 2019 (Table 1). A total of 990 clinically healthy live birds (86.8% of birds tested) were caught and sampled by ornithologists during regular ringing actions. The sampling was performed under the framework of the Polish national avian influenza monitoring program. The remaining 151 sampled birds (13.2%) had been found dead and were specimens that did not present any clinical signs of infection and died accidentally due to injuries caused by collisions with glass, power lines, and vehicles and were submitted for diagnostic purposes by ornithological stations and bird rehabilitation centers to the laboratory of the Department of Poultry Diseases of the National Veterinary Research Institute. All those birds were categorized into three groups of dietary preference (animal-based, mixed, and plant-based) based on the main food items known to be preferred by particular species of bird. Birds species were also categorized by preferred habitat (aquatic or terrestrial) and movement pattern (migratory or sedentary). All information about dietary preferences, habitats, and movement patterns was taken from the Birds of the World webpage^82^.

### Samples

Oropharyngeal swabs were collected from 1053 birds, cloacal swabs from 88 birds, and within this total, both oropharyngeal and cloacal swabs were taken from 60 birds. The samples were collected with the use of two types of swab: dry swabs from dead birds and swabs with a commercial transport system from live ones (ESwab Collection and Transport System, Copan Diagnostic, Murrieta, CA, USA). Each dry swab was placed into a tube with 500 µl of phosphate-buffered saline solution (PBS) and centrifuged at 20,000×*g* for 60 s before DNA isolation. The DNA of samples collected from live birds by swabs with the commercial transport system was extracted directly from the transport medium that was centrifuged at 20,000×*g* for 60 s. The DNA was extracted from 200 µl of sample using a QIAamp DNA Mini Kit (Qiagen, Hilden, Germany) following the manufacturer’s recommendations. The extracted DNA was frozen immediately and stored at − 20 °C for further analysis.

### PCR assay

We used a modified and optimized conventional PCR with primers located within the 16S ribosomal RNA (16S rRNA) as previously described by Lierz^83^. Briefly, the PCR mixture reaction consisted of 2.5 μl of 10 × PCR buffer, 1 μl of dNTPs (10 μM) (dATP, dCTP, dGTP, and dTTP), 0.5 μl of MgCl_2_ (1.5 mM), 1 μl of each primer (10 μM), 0.3 μl of OptiTaq polymerase (2 U, EURx, Gdańsk, Poland), 4 μl of Q solution (Qiagen, Hilden, Germany), 12.7 μl of water and 2 μl of the DNA sample. The following thermal cycling parameters were applied: 94 °C for 4 min, followed by 40 cycles of 94 °C for 30 s, 60.8 °C for 1 min, and 68 °C for 1 min, and a final extension of 68 °C for 10 min. The reaction was performed in a TPersonal thermocycler (Biometra, Goettingen, Germany). Five μl of each amplified product was separated by electrophoresis on a 2% agarose gel stained with ethidium bromide and visualized with ultraviolet light. MG and MS real-time PCR assays according to Raviv and Kleven^84^ were also performed in samples obtained from *Mycoplasma* spp.-positive birds.

### DNA sequencing and phylogenetic analysis

The phylogenetic analysis was performed using representative samples selected from different species of wild birds. Selected PCR products were sent for sequencing by the Sanger method to the Genomed laboratory (Warsaw, Poland). Each amplification product was sequenced in both directions with the forward and reverse amplification primers. Raw sequence data was analyzed with the FinchTV 1.4.0 software (Geospiza, Inc.; Seattle, WA, USA). High-quality sequences of 59 oropharyngeal swab samples and 7 cloacal swab samples were analyzed and then assembled using MEGA X 10.1 software^85^. Identification and analysis were carried out using the basic local alignment search tool (BLAST) algorithm. Closely related sequences of *Mycoplasma* spp. were downloaded and alignments of individual target sequences were constructed by the ClustalW algorithm implemented in MEGA X. The 16S rRNA-based phylogenetic tree (909 bp) was constructed with the maximum likelihood method and general time reversible model with Gamma distribution and 1000 bootstrap value in MEGA X software. Novel data for tested sequences of *Mycoplasma* spp. were deposited in the GenBank database under accession numbers MT358568–MT358581, MT358584–MT358587, MT367875–MT367892, MT367903-MT367913, MT374119–MT374126, MT374249–MT374256, MT549663, and MT678840–MT678841.

### Statistics

The prevalence of *Mycoplasma* spp. in wild birds was calculated as the proportion of *Mycoplasma*-positive birds to the total number of birds examined with 95% Wilson’s confidence intervals (95% CI). The relationships between results of the PCR and categorical explanatory variables including diet type (animal-based, mixed, or plant-based), type of habitat preferred (aquatic or terrestrial) and movement pattern (migratory or sedentary) were examined using the Fisher exact test in the *rcompanion* package version 2.3.26^86^. The *dplyr* package version 1.0.2 was used for data manipulation^87^ and the *ggplot2* package version 3.3.2 was used for result visualization^88^. All statistical calculations were performed using R version 4.0.3^89^.

### Ethics declarations

The swab sampling of live birds was performed for the purpose of avian influenza monitoring that is carried out by the laboratory of the Department of Poultry Diseases of the NRVI and no ethical permission was needed. Swabbing was by authorized and experienced ornithologists according to the Guidelines to the Use of Wild Birds in Research^90^.

## Supplementary Information

Supplementary Dataset.
